# Bilateral External Ventricular Drain Placement and Intraventricular Irrigation Combined with Concomitant Serial Prone Patient Positioning: A Novel Treatment for Gravity-Dependent Layering in Bacterial Ventriculitis

**DOI:** 10.7759/cureus.1175

**Published:** 2017-04-18

**Authors:** Andrew K Chan, Harjus S Birk, John K Yue, Ethan A Winkler, Michael W. McDermott

**Affiliations:** 1 Department of Neurological Surgery, University of California, San Francisco

**Keywords:** bacterial ventriculitis, external ventricular drain, gravity-dependent layering, intraventricular irrigation, neurosurgical intervention, outcomes, serial prone positioning, ventriculoperitoneal shunt

## Abstract

A feared complication of ventricular access for drainage or shunting is ventriculitis. Early diagnosis and treatment is vital to prevent morbidity and mortality. Efficacy of directed antibiotic therapy in ventriculitis is limited by increasing multidrug resistant microorganisms and insufficient systemic antibiotic absorption into the cerebrospinal fluid. Treatment may involve intravenous and/or intrathecal antibiotics as well as external ventricular drainage.

We present the first case report suggesting a potential role of a novel technique – direct ventricular catheter-mediated continuous saline irrigation and serial prone patient positioning – to treat a fulminant bacterial ventriculitis. This novel technique promotes egress of purulence from the ventricles and may result in more rapid control of intraventricular infectious burden.

## Introduction

Ventriculitis is a feared complication associated with cerebrospinal fluid (CSF) diversion and invasive intracranial pressure monitoring [1]. A large-scale retrospective review of 410 patients who had undergone external ventricular drain (EVD) placement revealed a 10.2% incidence of ventriculitis [2]. Its incidence ranges from <5% to 20%, with variability due to non-standard criteria for the diagnosis of ventriculitis.

First-line empiric treatment for ventriculitis includes a third generation cephalosporin in addition to vancomycin [3]. Unfortunately, the efficacy of present treatment paradigms is limited by antibiotic resistance and inadequate concentration of systemically administered antibiotic in CSF. For instance, it has been reported that vancomycin concentrations in the CSF following intravenous (IV) administration are often insufficient [4]. Unlike other surgical site infections where irrigation and drainage systems can be used to promote resolution of infection [5], the use of irrigation in the ventricular system has not been described for treatment of infection. Past experience with managing cases of intracranial hypotension has shown that intrathecal infusion of saline is tolerated without producing pain or neurologic symptoms and/or signs [6]. Based on prior experience and literature [7], we felt that the use of an irrigation and drainage system using saline for a severe case of bacterial ventriculitis with gravity-dependent layering in the ventricular system of pus was safe.

Herein, we report the case of 48-year-old man with hydrocephalus and a history of numerous ventriculoperitoneal shunt (VPS) placements and revisions. He developed ventriculitis after undergoing VPS placement and required removal of the VPS and placement of temporary CSF diversion. We present a novel technique of catheter-mediated direct continuous saline irrigation into the ventricle paired with intrathecal antibiotic administration and serial prone positioning to promote ventricular drainage.

## Case presentation

**Figure 1:**
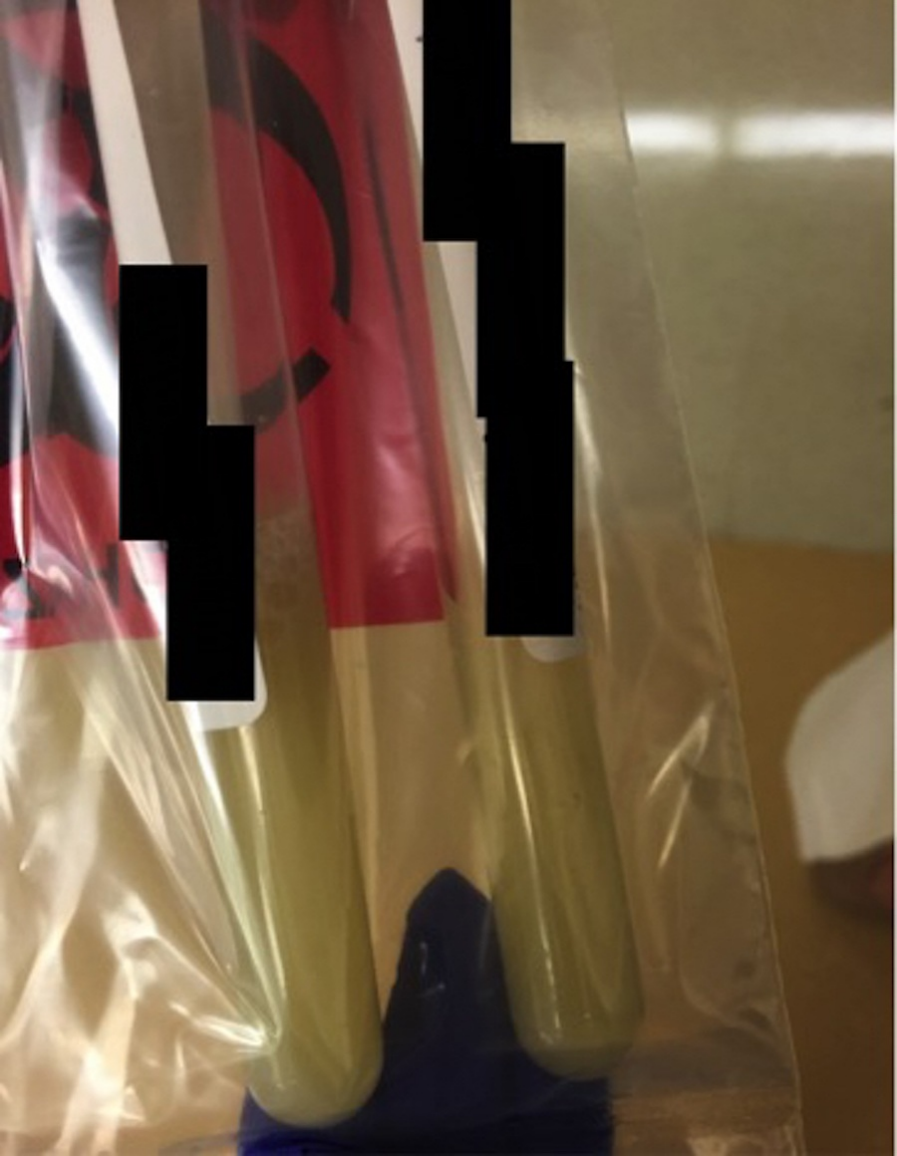
On treatment day 1, the patient’s shunt was tapped revealing grossly purulent fluid

The patient is a 48-year-old man who presented with recurrent, worsening symptoms of hydrocephalus following failed endoscopic third ventriculostomy. He subsequently underwent placement of a right frontal VPS. On post-operative day 4, the patient developed a high fever and altered mental status and underwent a shunt tap revealing grossly purulent fluid (Figure 1). Ultimately, this culture demonstrated E. coli resistant to trimethoprim-sulfamethoxazole (MIC > 4). He was brought immediately for shunt removal and EVD placement and was placed on broad-spectrum antibiotic coverage with IV meropenem 2 g every eight hours and IV vancomycin with goal trough 15-20 mg/dl.

At this time, we provided a novel treatment strategy for the patient that involved the following:

1. Placement of bilateral (right frontal, left frontal) EVDs.

2. Preservative-free normal saline was instilled in the right frontal EVD at 20 cc/hr with the left frontal EVD left open at 2 cm H2O.

3. Intraventricular gentamicin 8 mg daily was administered with both EVDs clamped for one hour after administration of the gentamicin.

4. Gentamicin to be administered intrathecally daily until three to five days after culture clearance.

5. Serial prone positioning for 30 minutes every three hours was carried out.

6. Care was taken to pad all pressure points well and a standard face cushion was applied which was identical to the cushion utilized in prone positioned cases in the operating room.

**Figure 2:**
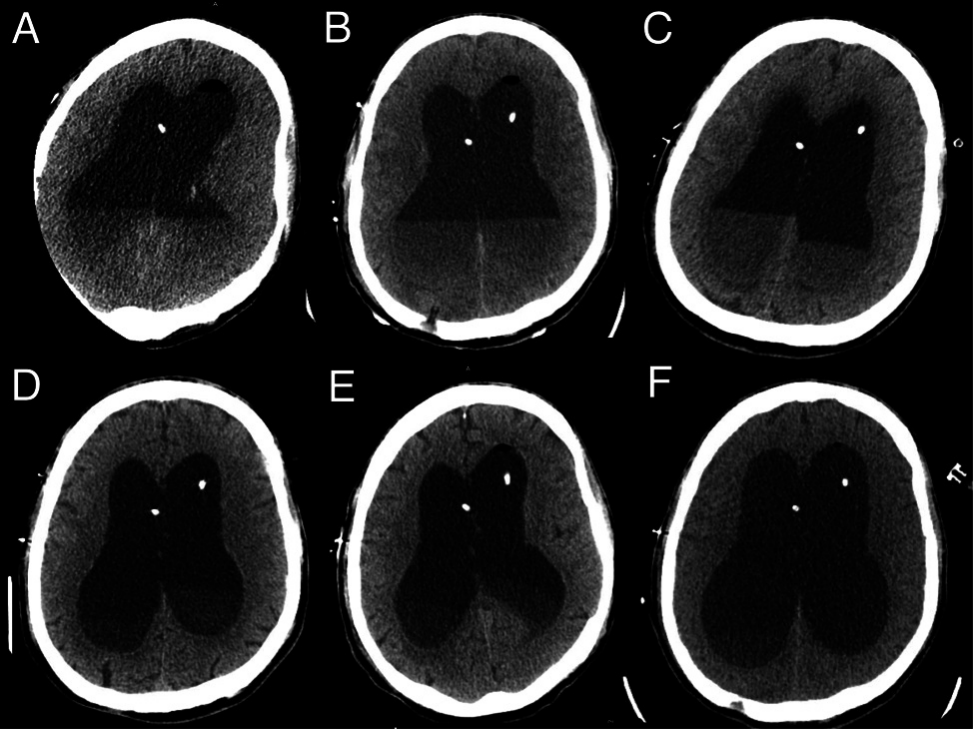
Axial non-contrast computed tomography (CT) cuts on treatment day 1, 2, 3, 7, 10, and 14 (A-F, respectively)

**Figure 3:**
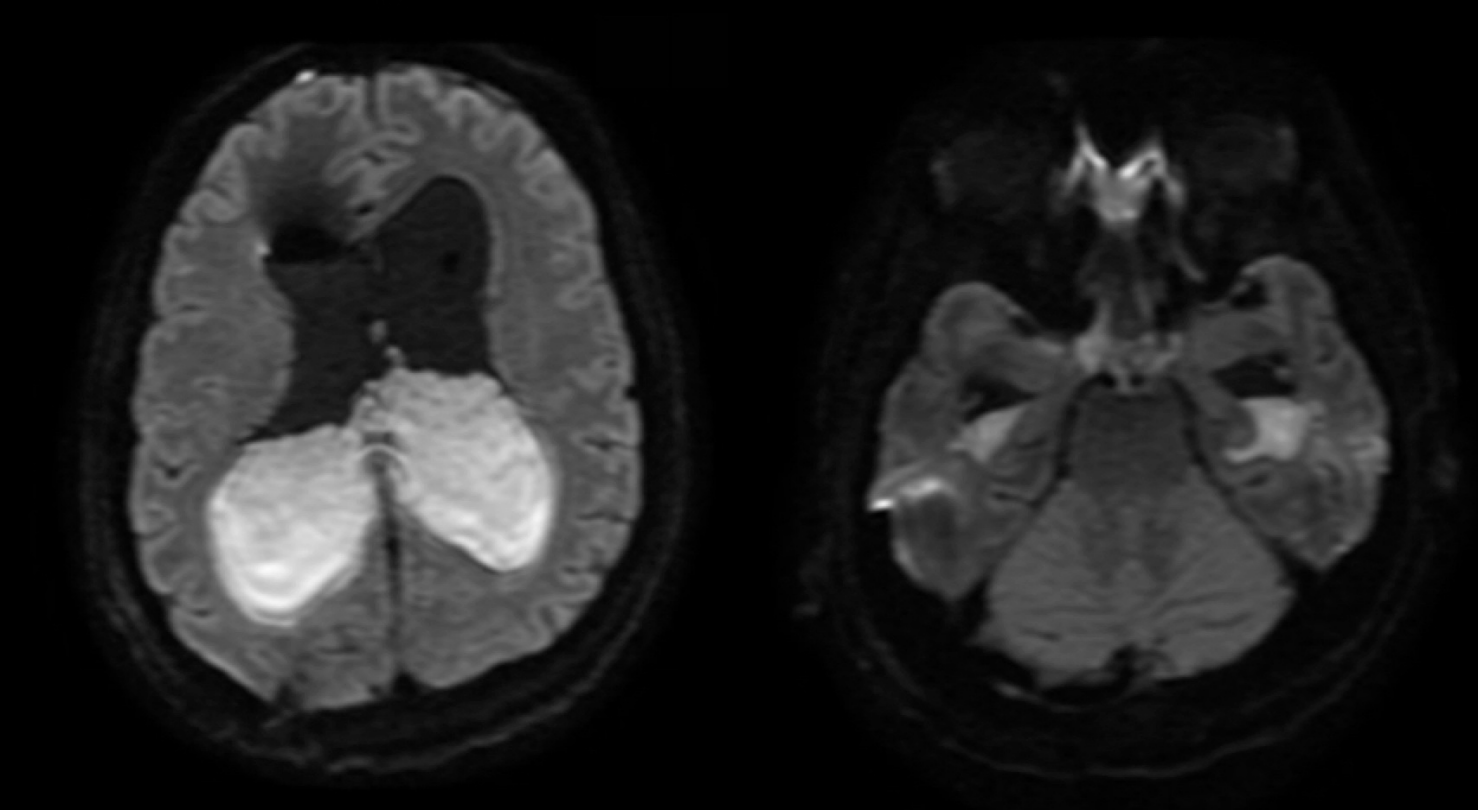
Magnetic resonance imaging (MRI) obtained (axial diffusion weighted imaging) at Day 4 of infection was notable for extensive infection with ventriculitis and basilar meningitis with diffuse subarachnoid pus throughout the basilar cisterns

At the outset of therapy, a computed tomography (CT) scan of the brain was obtained (Figure 2A, Day 1 of treatment) revealing layering purulence in the ventricular system. Given that the senior author had not previously seen this magnitude of purulence, it was decided to place the second left frontal EVD at this time (Day 1 of treatment). Indeed, magnetic resonance imaging (MRI) obtained at Day 4 of infection was notable for extensive infection with ventriculitis and basilar meningitis with diffuse subarachnoid pus throughout the basilar cisterns (Figure 3, Day 4 of treatment).

**Figure 4:**
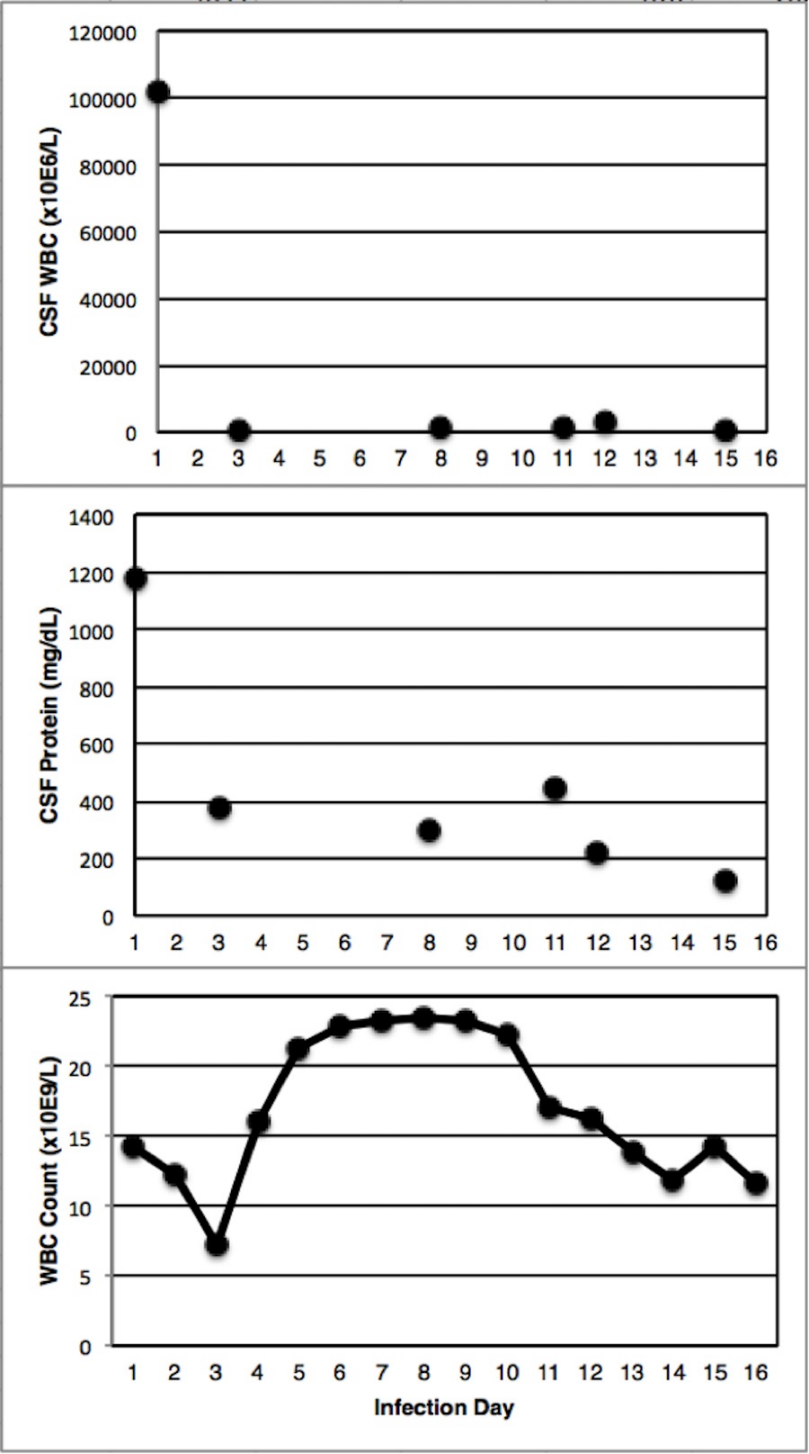
Trends of CSF WBC (top panel), CSF protein (middle panel), and systemic WBC count (bottom panel) over the course of treatment CSF: Cerebrospinal fluid; WBC: White blood cell.

Subsequent CT scans on Day 2, Day 3, Day 7, Day 10, and Day 14 of treatment (Figures 2B-2F) revealed rapid clearance of purulent material. This technique was discontinued when the CSF was clear and non-turbid. CSF cultures and cell count were obtained to monitor response to therapy. The response to treatment was rapid: by Day 2, CSF white blood cell (WBC) count improved from 102,000 to 720 and protein improved from 1180 to 376. CSF WBC counts, protein, and systemic WBC counts trends over the infection course are presented in Figure 4.

The patient improved clinically and CSF cultures were negative on two consecutive checks (Day 11 and 13) so the bilateral EVD were removed and a new shunt was implanted on Day 14. IV ceftriaxone was ultimately continued for a total of 21 days. The patient had no untoward effects from his bilateral EVD placement, intrathecal antibiotic administration, or serial prone positioning.

## Discussion

Bacterial ventriculitis is linked to significant morbidity and mortality following shunt surgery. Rapid drainage of purulent material may be beneficial in curtailing inflammation. Indeed, insufficient drainage may raise intracranial pressure and exacerbate intracranial infection [8]. This is the first case report to demonstrate the feasibility of serial prone positioning paired with catheter-guided continuous saline irrigation directly into the ventricles with prone positioning to flush sediment from the ventricles to assist in the treatment of ventriculitis.

There have been previous reports of intraventricular irrigation utilized to treat ventriculitis. A case report by Wada, et al. in 2000 reported a successful use of multiple intraventricular lavages for the treatment of post-traumatic ventriculitis in a 44-year-old male following traumatic brain injury [9]. In addition, a case report by Tabuchi and Kadowaki reported two cases of ventriculitis and progressive hydrocephalus after shunt infection successfully treated by neuroendoscopic septostomy in combination with intraventricular irrigation through a single burr hole [10]. Although surgical intervention is not a standard first-line treatment for ventriculitis, the authors highlight the feasibility of neuroendoscopic surgery in the management of this condition. We advocate the addition of serial prone patient positioning to prevent the sequestration of purulent material in the gravity-dependent portion of the lateral ventricles. This simple technique promotes rapid egress of the persistent purulent material layering out of the ventricle and thus expeditiously decreasing infectious burden. The efficacy of the technique in this case was confirmed by CT imaging, CSF protein levels, and WBC counts.

## Conclusions

This is the first report of the use of intraventricular catheter-mediated continuous saline irrigation paired with intraventricular antibiotic administration and serial prone patient positioning in the treatment of ventriculitis. Our case report demonstrates the safety and efficacy of this technique.
